# Xenbase: key features and resources of the *Xenopus* model organism knowledgebase

**DOI:** 10.1093/genetics/iyad018

**Published:** 2023-02-09

**Authors:** Malcolm Fisher, Christina James-Zorn, Virgilio Ponferrada, Andrew J Bell, Nivitha Sundararaj, Erik Segerdell, Praneet Chaturvedi, Nadia Bayyari, Stanley Chu, Troy Pells, Vaneet Lotay, Sergei Agalakov, Dong Zhuo Wang, Bradley I Arshinoff, Saoirse Foley, Kamran Karimi, Peter D Vize, Aaron M Zorn

**Affiliations:** Xenbase, Division of Developmental Biology, Cincinnati Children’s Hospital Medical Center, Cincinnati, OH 45229, USA; Xenbase, Division of Developmental Biology, Cincinnati Children’s Hospital Medical Center, Cincinnati, OH 45229, USA; Xenbase, Division of Developmental Biology, Cincinnati Children’s Hospital Medical Center, Cincinnati, OH 45229, USA; Xenbase, Division of Developmental Biology, Cincinnati Children’s Hospital Medical Center, Cincinnati, OH 45229, USA; Xenbase, Division of Developmental Biology, Cincinnati Children’s Hospital Medical Center, Cincinnati, OH 45229, USA; Xenbase, Division of Developmental Biology, Cincinnati Children’s Hospital Medical Center, Cincinnati, OH 45229, USA; Xenbase, Division of Developmental Biology, Cincinnati Children’s Hospital Medical Center, Cincinnati, OH 45229, USA; Xenbase, Division of Developmental Biology, Cincinnati Children’s Hospital Medical Center, Cincinnati, OH 45229, USA; Xenbase, Department of Biological Sciences, University of Calgary, Calgary, AB T2N 1N4, Canada; Xenbase, Department of Biological Sciences, University of Calgary, Calgary, AB T2N 1N4, Canada; Xenbase, Department of Biological Sciences, University of Calgary, Calgary, AB T2N 1N4, Canada; Xenbase, Department of Biological Sciences, University of Calgary, Calgary, AB T2N 1N4, Canada; Xenbase, Department of Biological Sciences, University of Calgary, Calgary, AB T2N 1N4, Canada; Xenbase, Department of Biological Sciences, University of Calgary, Calgary, AB T2N 1N4, Canada; Department of Biological Sciences, Carnegie Mellon University, Pittsburgh, PA 15213, USA; Xenbase, Department of Biological Sciences, University of Calgary, Calgary, AB T2N 1N4, Canada; Xenbase, Department of Biological Sciences, University of Calgary, Calgary, AB T2N 1N4, Canada; Xenbase, Division of Developmental Biology, Cincinnati Children’s Hospital Medical Center, Cincinnati, OH 45229, USA

**Keywords:** Xenbase, *Xenopus*, model organism database, genomics, ontology

## Abstract

Xenbase (https://www.xenbase.org/), the *Xenopus* model organism knowledgebase, is a web-accessible resource that integrates the diverse genomic and biological data from research on the laboratory frogs *Xenopus laevis* and *Xenopus tropicalis*. The goal of Xenbase is to accelerate discovery and empower *Xenopus* research, to enhance the impact of *Xenopus* research data, and to facilitate the dissemination of these data. Xenbase also enhances the value of *Xenopus* data through high-quality curation, data integration, providing bioinformatics tools optimized for *Xenopus* experiments, and linking *Xenopus* data to human data, and other model organisms. Xenbase also plays an indispensable role in making *Xenopus* data interoperable and accessible to the broader biomedical community in accordance with FAIR principles. Xenbase provides annotated data updates to organizations such as NCBI, UniProtKB, Ensembl, the Gene Ontology consortium, and most recently, the Alliance of Genomic Resources, a common clearing house for data from humans and model organisms. This article provides a brief overview of key and recently added features of Xenbase. New features include processing of *Xenopus* high-throughput sequencing data from the NCBI Gene Expression Omnibus; curation of anatomical, physiological, and expression phenotypes with the newly created *Xenopus* Phenotype Ontology; *Xenopus* Gene Ontology annotations; new anatomical drawings of the Normal Table of *Xenopus* development; and integration of the latest *Xenopus laevis* v10.1 genome annotations. Finally, we highlight areas for future development at Xenbase as we continue to support the *Xenopus* research community.

## Introduction

*Xenopus* has a great impact as a biomedical model because of its unique experimental advantages, cost-effectiveness, and close evolutionary relationship to mammals (Blackburn and Miller 2019; Hwang *et al.* 2019; Kakebeen and Wills 2019). The remarkable utility of *Xenopus* continues to reveal new insights into an incredibly diverse suite of biological domains. Many of the principles first discovered in *Xenopus*, particularly in the areas of developmental and cell biology, neuroscience, pharmacology, genomics, and, more recently, disease modeling continue to have a lasting impact on understanding human health (Gurdon *et al.* 1958; Smith *et al.* 1990; Takahashi and Yamanaka 2006; McLin *et al.* 2007; Ferrell *et al.* 2011 ; Harland and Grainger 2011; Grainger 2012; Nenni *et al.* 2019).

Two *Xenopus* species are commonly used in biomedical research. The original *Xenopus laevis* is allotetraploid with larger embryos, whereas the more recently adopted *Xenopus tropicalis* is diploid and has slightly smaller embryos but is excellent for genetics. Both species share several experimental advantages. Thousands of synchronously developing eggs allow the production of cell extracts that are invaluable for characterizing the biochemical mechanisms of the cell cycle and cytoskeleton (Ferrell *et al.* 2011; Harland and Grainger 2011). *Xenopus* embryos are ideal for studying gene function during embryogenesis by simple microinjection of mRNAs, antisense morpholinos, or genome editing constructs, because a well-defined cell fate map allows easy tissue-restricted manipulation. CRISPR/Cas9 gene editing is very effective in *Xenopus*, both for transient biallelic mutations in F0 embryos (*X. laevis* and *X. tropicalis*) (Aslan *et al.* 2017) and for multigenerational genetics in *X. tropicalis* (Tandon *et al.* 2017; Naert and Vleminckx 2018; Kakebeen and Wills 2019; Nakayama *et al.* 2020; Exner and Willsey 2021). The growing number of transgenic and mutant lines increasingly allows precise, temporal-spatial manipulation of gene expression and function. Rapid *Xenopus* transgenics allows for studying genomic *cis*-regulation (Fish *et al.* 2012) and the evolutionary position of *Xenopus* places it as an excellent model for comparative genomics and screening mammalian enhancers (Sun *et al.* 2014). The large and robust *Xenopus* cells are well suited for in vivo imaging, micro-dissection, and embryonic organ culture, providing ample material for quantitative biochemical and single-cell genomic analyses. Externally developing *Xenopus* larvae are also particularly suited to drug and toxin screening, as cell-soluble small molecules can be added to the culture media (Wuhr *et al.* 2014). Hundreds of physiologists and toxicologists around the world also use *Xenopus* oocytes as a single-cell expression system to examine the activity of human receptors and membrane channels (Kvist *et al.* 2011). Finally, *Xenopus* is increasingly used to model human diseases such as craniofacial malformations, congenital heart disease, neuropathies, epilepsy, and cancer, to name a few (Cross and Powers 2009; Bell *et al.* 2011; Fakhro *et al.* 2011; Bonnard *et al.* 2012; Yoon *et al.* 2012; Nenni *et al.* 2019; *Xenopus community 2020*).

Xenbase, the *Xenopus* model organism knowledgebase (Fortriede *et al.* 2020), was founded in 2000. Over the last 2 decades, Xenbase has had a transformative impact on *Xenopus* research and on the visibility of *Xenopus* data in the broader scientific community. The integration of genomic and biological data has driven the greatest leap forward in biomedical research since the advent of recombinant DNA. As a result, model organism knowledgebases (MOKs) like Xenbase have become standard, necessary resources to translate the research from animal models into a meaningful biological synthesis that can impact human health. Xenbase is the only central repository that integrates the vast and diverse body of information from *Xenopus* research, supporting over 500 research labs from around the world. Our relational database and user-friendly, intuitive interface allow investigators to quickly interrogate and link different types of data such as genomics, expression, function, and phenotypes in ways that would otherwise be difficult, time consuming, or impossible. Xenbase implements the FAIR data management principles (FAIR Data Principles 2022) that aim to make data findable, accessible, interoperable, and reusable (Wilkinson *et al.* 2016). Xenbase enhances the value of *Xenopus* data through high-quality curation, ontology development, data integration, bioinformatics tools optimized for *Xenopus* experiments, and linking *Xenopus* data to humans and other model organisms. Xenbase plays an indispensable role in making *Xenopus* data accessible to the broader biomedical community by continually providing annotated data updates to many organizations such as the NCBI and other MOKs.

In this article, we will summarize the current status of Xenbase and recent additional features, including support for the newest genomes, high-throughput sequencing data (RNA-seq and ChIP-seq), new phenotype and disease modeling, ontology development, and Gene Ontology curation. We will provide an overview of our semi-automated curation pipeline that allows us to curate the ∼1,000 new *Xenopus* research papers published each year. Finally, we discuss recent efforts for Xenbase to join the Alliance of Genome Resources making *Xenopus* data even more accessible and interoperable for the research community.

## Overview of Xenbase content and usage

**Fig. 1.**
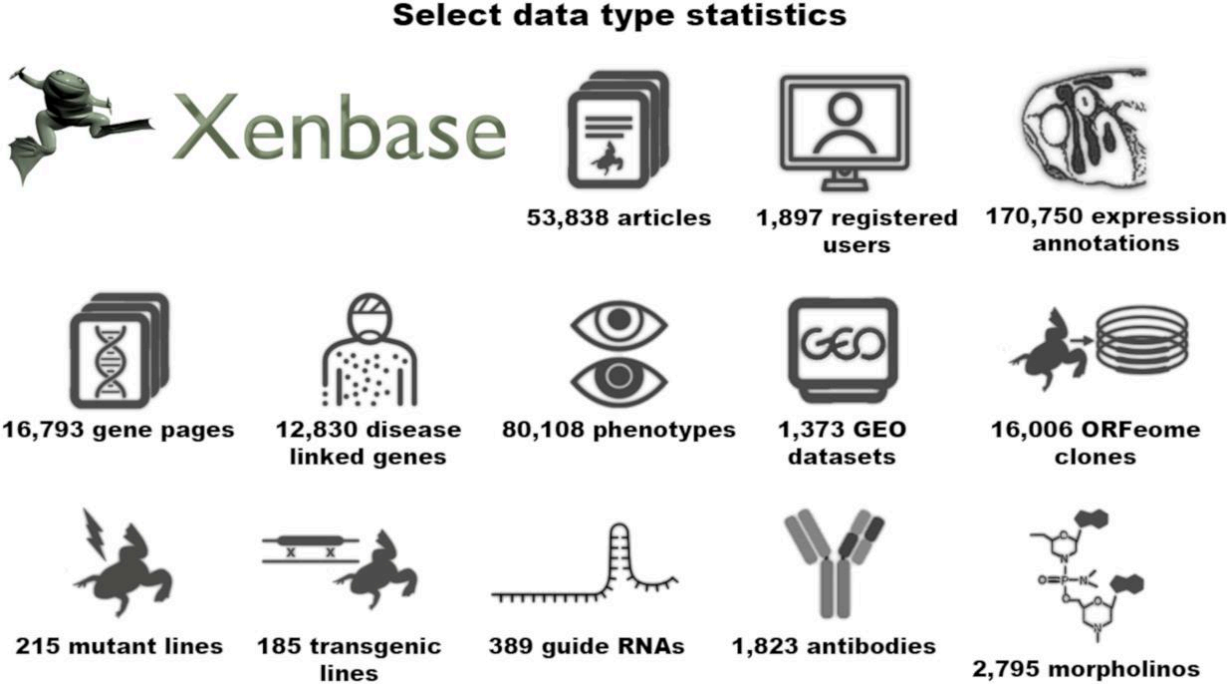
Xenbase data summary statistics. This infographic shows statistics for several key Xenbase data types.

Xenbase contains data from over 53,000 published papers from PubMed integrated with all the *Xenopus* DNA, RNA, and protein sequences from GenBank and Ensembl, including both *X. laevis* and *X. tropicalis* genomes. Xenbase uses a semi-automated curation workflow to triage and curate papers annotating gene function, expression, experimental phenotypes, disease associations, transgenes, mutants, and research reagents such as antibodies, antisense morpholino oligos, and CRISPR guide RNAs. Figure 1 provides a graphical summary of the current data content. To annotate these data, Xenbase developed several ontologies including the *Xenopus* anatomy ontology (XAO) (Segerdell *et al.* 2013) and *Xenopus* phenotype ontology (XPO) (Fisher *et al.* 2022). Xenbase hosts a BLAST server dedicated to *Xenopus* sequences and JBrowse (Buels *et al.* 2016) to support the *Xenopus* genomes. Xenbase also curates and processes all the *Xenopus* RNA-seq and ChIP-seq data available in the NCBI GEO short-read archive allowing us to computationally derive over 80,000 experimental gene expression phenotype annotations. Xenbase collaborates with the *Xenopus* stock centers, including the National *Xenopus* Resource (NXR, RRID:SCR_013731), the European *Xenopus* Resource Centre (EXRC, RRID:SCR_007164) (Pearl *et al.* 2012), and the National BioResource Project (NBRP) in Japan (Yamazaki and Sugawara 2009), to maintain a catalog of *Xenopus* transgenic lines, mutants, and strains and information on their availability (Horb *et al.* 2019). In addition, Xenbase contains invaluable static content for researchers, including protocols, anatomy resources, and educational material, and serves as a communications focal point for the *Xenopus* research community.

Xenbase currently has over 1,800 registered users representing about 500 *Xenopus* laboratories around the world. Google Analytics reports over 4,000 user sessions per week, over 80% of which are return visitors. These are our core user base who visit Xenbase daily to weekly and spend, on average, over 7 minutes viewing 5–8 pages. The most viewed features are Gene Pages, the landing page, the Anatomy Atlas, Genome Browsers, and BLAST. Our content is kept up to date with periodic synchronization with other resources such as NCBI, Ensembl, and UniProt. Xenbase also provides data exchange files for other sites, including the NCBI, UniProt, and the Gene Ontology Consortium. The data files stored on the Xenbase data download site are accessed by ∼120 Unique IP addresses per month, which ingest an average of 68 GB of data.

## Navigating Xenbase

**Fig. 2.**
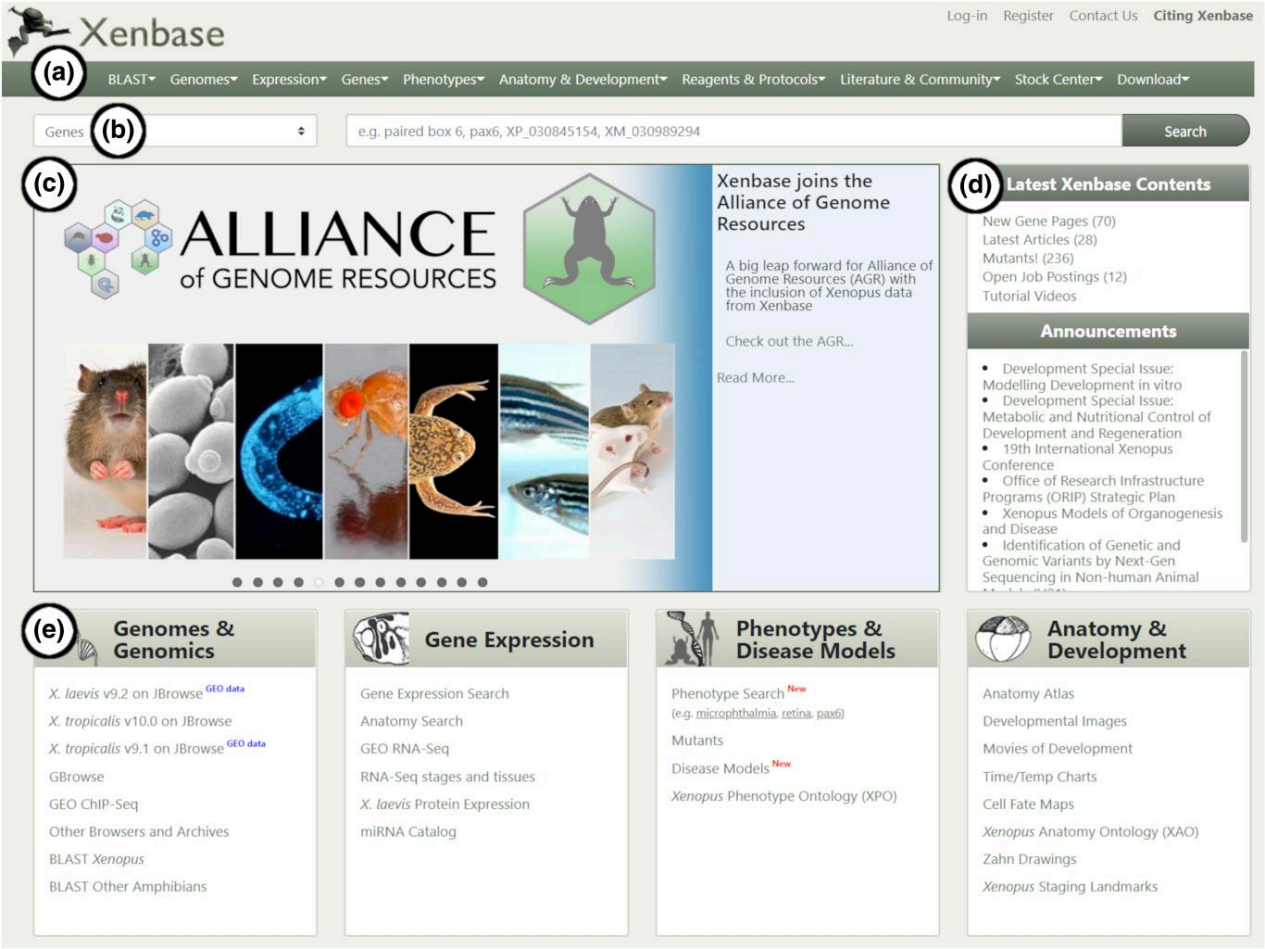
Xenbase landing page. a) Header menu dropdowns. b) Quick search bar. c) Latest news slideshow. d) Sidebar with summary of recently added data and recent community announcements. e) Tiled view of key topic categories and quick access links.

The Xenbase landing page (https://www.xenbase.org/) has been streamlined and designed to allow intuitive navigation with multiple redundant paths for users to interact with the content through different user-preferred workflows (Fig. 2). Key resources are provided in “tiles” representing 4 core categories (Fig. 2e): “Genome & Genomics,” where the various available genome builds are available for download, searching, or browsing; “Gene Expression,” which links to our expression search and GEO data; “Phenotypes & Disease Models,” which links to our phenotype search interface and mutant line information; “Anatomy & Development,” which links to a number of our key anatomy resources, including the anatomy ontology and tables of development. Each of these is also available in the drop-down menus of the horizontal page header (Fig. 2a), which is persistent across all Xenbase pages. The header menus offer further links for Reagents & Protocols, Literature & Community, Stock Center, and Download. There is also a quick search bar that allows searching for a variety of data types (Fig. 2b). The quick search does not discriminate between *X. tropicalis* and *X. laevis* but the more dedicated search tools accessible through the landing page tiles or header drop-down menus do allow species-specific searches. The quick search defaults to genes so that a simple gene search is the easiest thing to perform from the landing page and leads directly to the relevant Gene Page—the main data portal on Xenbase.

## Xenbase gene pages

**Fig. 3.**
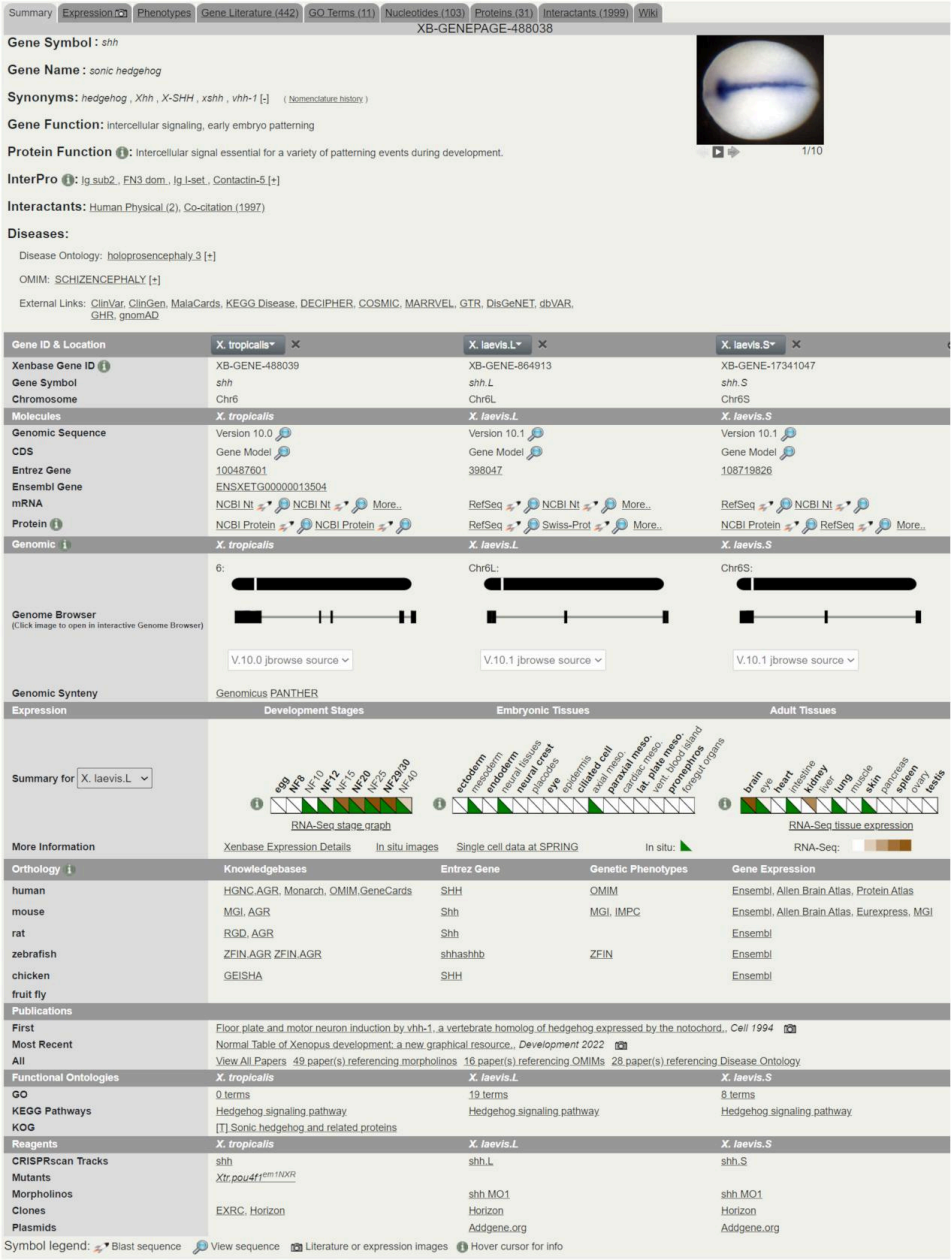
Xenbase gene page layout. The top section of the Gene Page shows a brief overview of relevant data and links to both more detailed Xenbase pages and external resources. Tabs switch to further gene-specific data on the relevant topics. The lower sections provide species-specific data for *X. tropicalis* and *X. laevis* including graphical summaries of genomic position and gene expression. This example is for the shh gene (https://www.xenbase.org/entry/XB-GENE-488038).

Data on Xenbase are organized primarily around Gene Pages that provide both a high-level summary and detailed information on both *X. laevis* and *X. tropicalis* genes (Fig. 3). Data are arranged in a tabbed manner, with the first tab being Summary data, followed by Expression, Phenotypes, Gene Literature, GO Terms, Nucleotides, Proteins, Interactants, and Wiki. These tabs lead to much more detailed information for each subject but the basic outline of the Summary tab is given below. This organization allows us to keep Gene Pages uncluttered.

*Xenopus laevis* is an allotetraploid due to an ancient hybridization event resulting in 1 set of 9 long (L) chromosomes and a second set of 9 short (S) chromosomes, so there are 2 *X. laevis* genes for each locus (with some exceptions), while there is a single gene for each locus found in *X. tropicalis* (Hellsten *et al.* 2010; Session *et al.* 2016). Thus, most Gene Pages contain 3 genes: one *X. tropicalis* as well as separate *X. laevis* L and S versions of each gene. The Gene Page summary tab provides the official gene symbol and name along with any synonyms and a link is provided to the nomenclature history Wiki, where all gene name changes are recorded. A summary of the gene/protein function is provided based on both UniProt and Xenbase curation, both as text descriptions and as a set of InterPro domains identified in the protein product of the gene. Shortcuts to physical interaction data from human orthologs and co-citation networks for the *Xenopus* genes are provided in the Interactants section; the co-citation graphs are gene–gene networks based on the co-occurrence of genes in articles. A list of disease terms is associated with the gene, either via phenotype curation in Xenbase or from associations from external sources such as Monarch or Online Mendelian Inheritance in Man (OMIM). We also provide link-outs to several other gene-centric disease resources such as ClinVar (Landrum *et al.* 2020), DECIPHER (Firth *et al.* 2009), and MARRVEL (Wang *et al.* 2017). These resources are linked using human ortholog IDs, either HUGO Gene Nomenclature Committee (HGNC) or NCBI Gene IDs, as provided by NCBI or using the gene symbol depending on what query inputs the resources accept.

The “Gene ID & Location” section specifies the chromosome, Xenbase XB-GENE-ID, and species- or subgenome-specific gene symbol. The “Molecules” section then provides links to the genomic, mRNA, and protein sequences as well as linking to the equivalent gene pages on NCBI (Entrez) and Ensembl. The “Genomic” section provides a brief schematic of the gene model structure with links to the JBrowse genome viewer. This section also provides links to synteny/orthology resources such as Genomicus (Nguyen *et al.* 2022) and PANTHER (Mi *et al.* 2019).

The “Expression” section of the summary tab shows a summary expression ribbon, where the species can be selected in a drop-down menu on the left-hand side. This ribbon shows both temporal and spatial expression from manual curation of in situ hybridization and/or RNA-seq data with key development stages and tissues selected from the XAO. Any expression annotations made to children of the upper-level XAO term will also be shown, so for example the eye section will count any annotations made to either the retina or the lens. A summary expression image is often included on Gene Pages such as in situ hybridization, with much more detailed data available on the expression tab. For more details on expression-related features, see the “Gene Expression Data” section below.

The “Orthology” section provides link-outs to a wide variety of external resources for orthologous genes from humans and several different model organisms. These resources cover phenotype and expression information as well as linking to the MOKs for each organism. The “Publications” section provides the latest and first publication in our corpus that mentions the gene of interest as well as links to fuller and more granular literature searches. The “Functional Ontologies” section has links to GO term associations from EBI's gene ontology annotation project (GOA) (Huntley *et al.* 2015) as well as the KEGG pathway (Kanehisa *et al.* 2017) and Eukaryotic Orthologous Groups (KOG) (Tatusov *et al.* 2003) functional classifications. The final section on the Summary tab, Reagents, links to predicted CRISPR target sites on our JBrowse instance, published experimental guide RNAs, antibodies, morpholinos, and ORFeome clones available from the EXRC. This section also provides link-outs to searches for clones and plasmids on external reagent supplier resources.

## *Xenopus* genomes

Xenbase is a source for the latest (pre-GenBank submission), current, and legacy assemblies of the *Xenopus* genomes. The genomes are available for browsing in our JBrowse instance, download from the Xenbase data download site (GFF3, GTF, and FASTA formats), and for sequence searching via both gene name/symbol searches, chromosome/scaffold coordinates, and the Xenbase BLAST module. We also provide files for viewing *Xenopus* genome data via a UCSC browser track hub.

In the last few years, new high-quality chromosome-level genome assemblies for both *X. laevis* and *X. tropicalis* have been generated by the International *Xenopus* Sequencing Consortium, led by the Rokhsar laboratory at the University of California at Berkeley (UCB). The current versions deposited in GenBank are v10 for *X. tropicalis* (Mitros *et al.* 2019) (BioProject:PRJNA577946, Refseq:GCF_000004195.4) and v10.1 for *X. laevis* (BioProject:PRJNA313213, RefSeq:GCF_017654675.1). *Xenopus tropicalis* v10 has 28,858 genes, of which 21,826 are protein coding. For *X. laevis* version 10.1, there are 44,456 genes, 34,476 of which are protein coding. Three groups (UCB, NCBI, and Ensembl) have independently annotated the genome assemblies. Since each group uses its own computational pipelines to predict gene models, there are often discrepancies in both gene model structure and gene nomenclature. To account for this, Xenbase provides all 3 annotations in JBrowse to allow a direct comparison. In addition, Xenbase is working to provide consensus gene models and include improved nomenclature coverage as provided previously for the version 9 builds of *X. laevis* and *X. tropicalis* (Karimi *et al.* 2018). When our consensus Xenbase GFFs are prepared, they will be available on the Xenbase data download site and JBrowse instance.

These latest genome builds, v10 for *X. tropicalis* and v10.1 for *X. laevis*, are now supported on Xenbase with integration into Gene Page content, representation on the Xenbase JBrowse instance, and availability on our BLAST servers. Several of the cataloged reagents associated with nucleotide sequences have been updated to reflect these latest genome builds, including morpholinos and guide RNAs which have all been aligned against the latest genomes and are represented in both the reagent resources and on tracks for JBrowse.

## Gene nomenclature

Xenbase is the official administrator of the *Xenopus* gene nomenclature. We work with the HUGO Gene Nomenclature Committee (HGNC), *Xenopus* and Vertebrate Gene Nomenclature committees, the National Center for Biotechnology Information (NCBI), researchers, and domain experts to establish and maintain up-to-date *Xenopus* nomenclature. Nomenclature guidelines are accessible at https://www.xenbase.org/entry/static/gene/geneNomenclature.jsp. *Xenopus* nomenclature follows that of the human ortholog, with *Xenopus* gene names and symbols in lowercase italics. Establishing orthology is an ongoing major undertaking for many international database resources (e.g. the Alliance of Genome Resources, UniProt, and EMBL-EBI), and we work with multiple analytical tools [e.g. FastOrtho (FastOrtho 2016), InParanoid (Persson and Sonnhammer 2022), OrthoFinder (Emms and Kelly 2019), ProteinOrtho (Lechner *et al.* 2011), SwiftOrtho (Hu and Friedberg 2019), SonicParanoid (Cosentino and Iwasaki 2019), PhylomeDB (Fuentes *et al.* 2022), and the DIOPT (Hu *et al.* 2011; DRSC Integrative … 2015) pipeline] to establish orthology between *Xenopus*, human, and other model organisms. Additionally, we collaborate with NCBI RefSeq genome curators to determine orthologous gene relationships, using a combination of phylogenetics, protein sequence similarity, and local synteny information from the latest genome assemblies to correct or apply appropriate names, including new gene names/symbols for “unnamed” and “uncharacterized” gene models. In cases where genes/gene families are amphibian specific, or are not found in humans or mammals, we consult the relevant nomenclature committees and domain experts and refer to published literature to ensure that the proposed “new” gene names and symbols do not conflict with existing nomenclature in other taxa.

Xenbase curators collaborate with researchers around the world to improve gene naming and gene annotation. We recently completed a major review of several amphibian immune system gene families (including classical and nonclassical MHC genes, interferons, and interleukins), and we named over 400 *Xenopus tropicalis* olfactory receptor genes, using the Mutual Maximum Similarity algorithm as described in Olender *et al.* (2020). The need for gene nomenclature updates also arises from newly published research [e.g. *Xenopus* potassium channel *kcnj* genes (Rangel-Garcia *et al.* 2021) and opsins (Bertolesi *et al.* 2020)], and in response to HGNC changes to human gene names (following the continual revision and characterization of human genes), and from analysis from other model organism database curators and NCBI RefSeq curators (who often identify non-mammalian gene family expansions). Recent updates from these sources include heat-shock proteins, several previously uncharacterized genes, and the nomenclature for mitochondrially encoded genes.

Moving forward, one of Xenbase's major goals is to improve gene nomenclature for *Xenopus* by assigning names to the many “uncharacterized” genes and replacing “LOC” gene symbols with more meaningful names (e.g. changed *LOC108700223* to *gpsm3*). Most newly assigned gene names and gene symbols, and those still under review, are tagged with a “provisional” suffix.

## Gene expression data

Xenbase gene expression data are available in several forms: normal expression assayed with in situ hybridization or immunohistochemical methods, normal expression assayed by high-throughput sequencing techniques such as RNA-Seq, and abnormal expression data from a variety of assay methods that will be covered in greater depth in the following “Phenotypes & Disease Models” section.

Gene expression data can be found through the Xenbase “Expression Search” interface that allows for complex multifactored searches; through Gene Page “Expression” tabs; or through images in articles with associated curations. RNA-seq expression data can be found in the GEO data section, described above.

For a thorough overview of available data on the expression of a gene, the Gene Page “Expression” tab offers the most variety and provides the following information: (1) an exhaustive list of anatomical locations from the XAO that have been annotated as showing expression of the gene of interest; (2) links to specific detailed stage series RNA-Seq data, from the Owens *et al.* (2016) study for *X. tropicalis,* and the 2016 Session *et al.* study for *X. laevis* (Session *et al.* 2016). Thumbnail profiles of these data are also provided, and a fuller version can be reached by clicking on the expression profiles, and the newly opened version also allows for the addition of custom genes to the visualization for comparative purposes; (3) link-outs to GEO profile expression data at the NCBI; (4) expression profiles from an older microarray stage series (Yanai *et al.* 2011); (5) for *X. laevis* adult, tissue expression data for specific tissues and organs are available, drawn from the Session *et al.* (2016) data; (6) a heatmap showing expression changes associated with classical dorsoventral specification manipulations from Ding *et al.* (2017); (7) expression images highlighted by the Xenbase curation team as good examples of expression of the gene of interest; (8) community-submitted images of expression of the gene of interest; (9) images from the published literature curated for expression of the gene of interest.

The other significant source of expression data in Xenbase is high-throughput RNA-Seq data from GEO processed through the Xenbase GEO pipeline. Xenbase continues to curate and process high-throughput datasets from the NCBI Gene Expression Omnibus (GEO) repository. The details of the pipeline for processing and integrating the GEO data into Xenbase are previously published (Fortriede *et al.* 2020), covering the method of describing experimental conditions, data processing, and the various visualization options. We are continuing to expand the types of assays we can process, most recently we have begun analyzing Assay for Transposase-Accessible Chromatin sequencing (ATAC-Seq) data. The system for curating the experimental details of the GEO series is extensively integrated with the new phenotype module both due to the overlap in functionality and to allow expression phenotypes to be derived from high-throughput data, see the Phenotype section for more details.

**Fig. 4.**
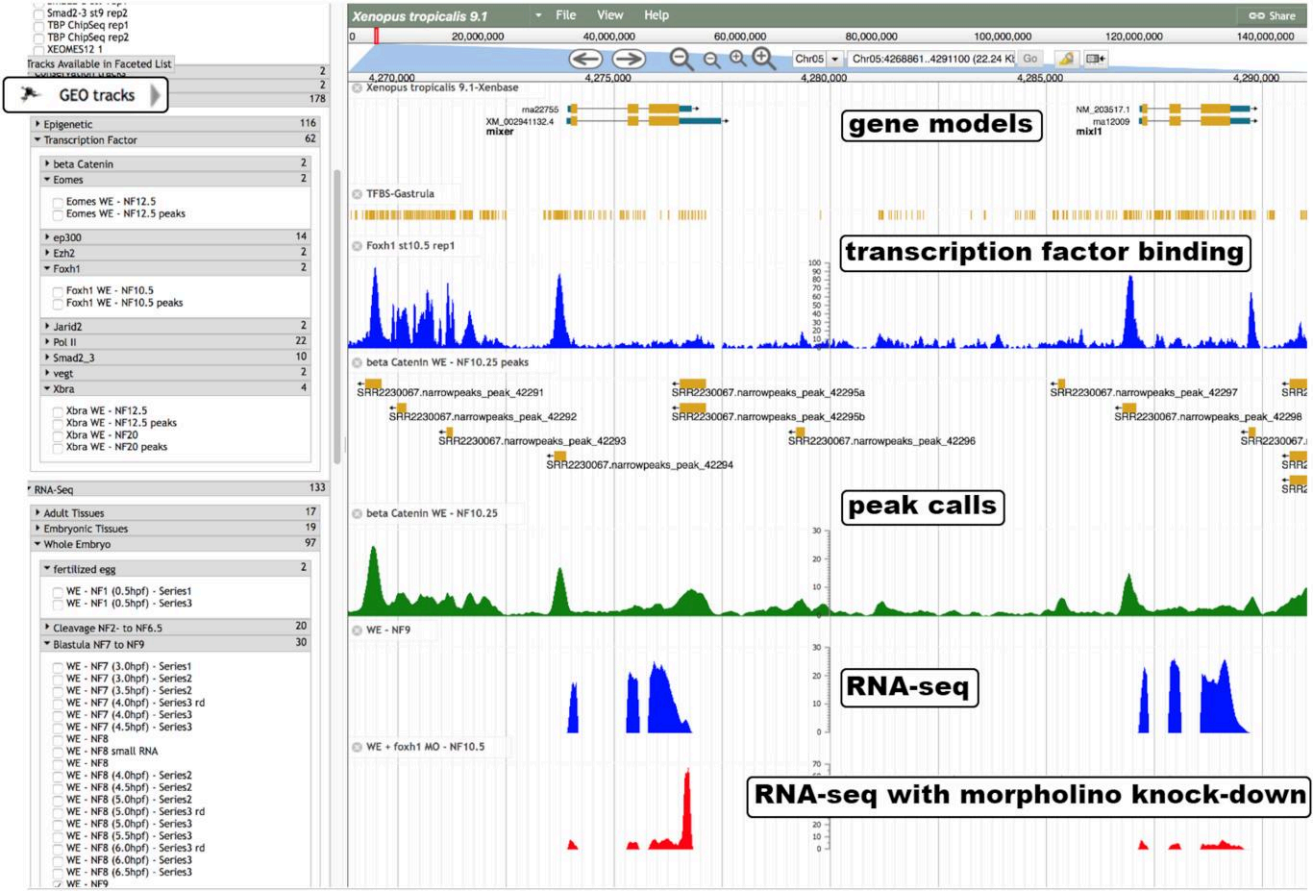
Track types available on Xenbase JBrowse. The Xenbase JBrowse instance provides many different track types, such as gene and transcript models, transcription factor binding site data, and RNA-seq data. To the left of the main display is the track selection interface which allows categories of tracks to be navigated and selected, also shown is the “GEO tracks” button which opens an alternative faceted browsing interface for selecting processed GEO tracks.

**Fig. 5.**
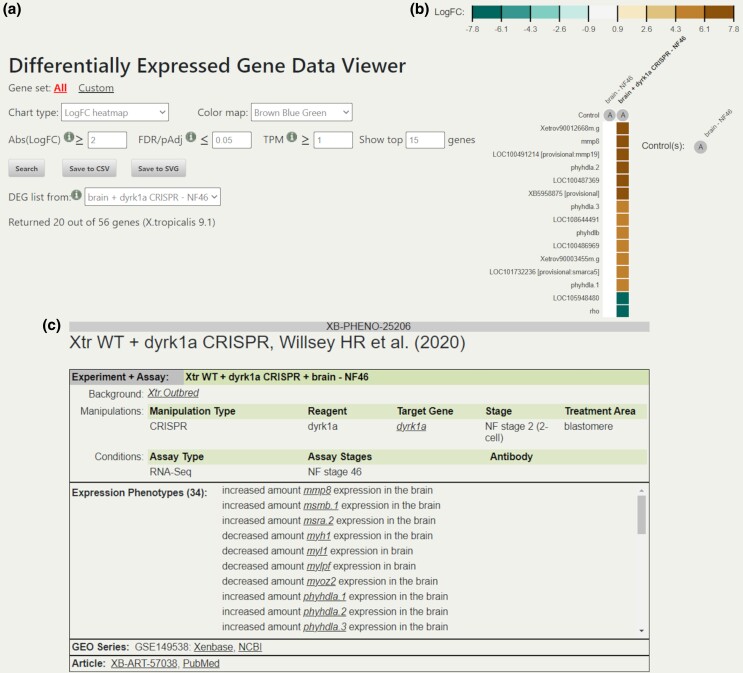
GEO expression data and phenotypes. a) GEO heatmap visualization parameter options as our automated expression pipeline, the values here are the same as those used to derive computational expression phenotypes. b) Heatmaps showing samples from a control brain and a brain from a CRISPR knockdown of *dyrk1a*. The color map is scaled to the highest absolute value of the maximum and minimum values of the genes displayed. c) An XB-PHENO page displaying part of the corresponding computationally derived expression phenotypes. This differs from the manual XB-PHENO page layout, see Fig. 6c, in the lack of accompanying image and additional links to the Xenbase and NCBI pages for the original GEO series and research article (Willsey *et al*. 2020). The phenotype data shown here are available at https://www.xenbase.org/entry/XB-PHENO-25206.

GEO datasets can be searched through the “GEO data@Xenbase” search interface providing searches on several criteria and an advanced search that allows combining criteria. We provide data in a variety of genome browser tracks for the Xenbase JBrowse module such as RNA-Seq and ChIP-Seq data, including ChIP peaks (Fig. 4). Within the JBrowse module, we have an additional method of searching or filtering GEO data, the GEO tracks faceted browser. We also provide GEO data visualizations and analyses including ranked lists of differentially expressed genes, heatmaps showing differential expression values and direct TPM (transcripts per million) counts (Fig. 5a), parallel coordinate plots, and hive plots. Further details on mining the GEO data on Xenbase to visualize expression can be found in Fortriede *et al.* (2020). We provide several tutorial videos showing how to find and extract data using the “GEO data@Xenbase” interface and the Xenbase JBrowse instance (https://www.xenbase.org/entry/static-xenbase/HowTo.jsp).

## Phenotypes and disease models

**Fig. 6.**
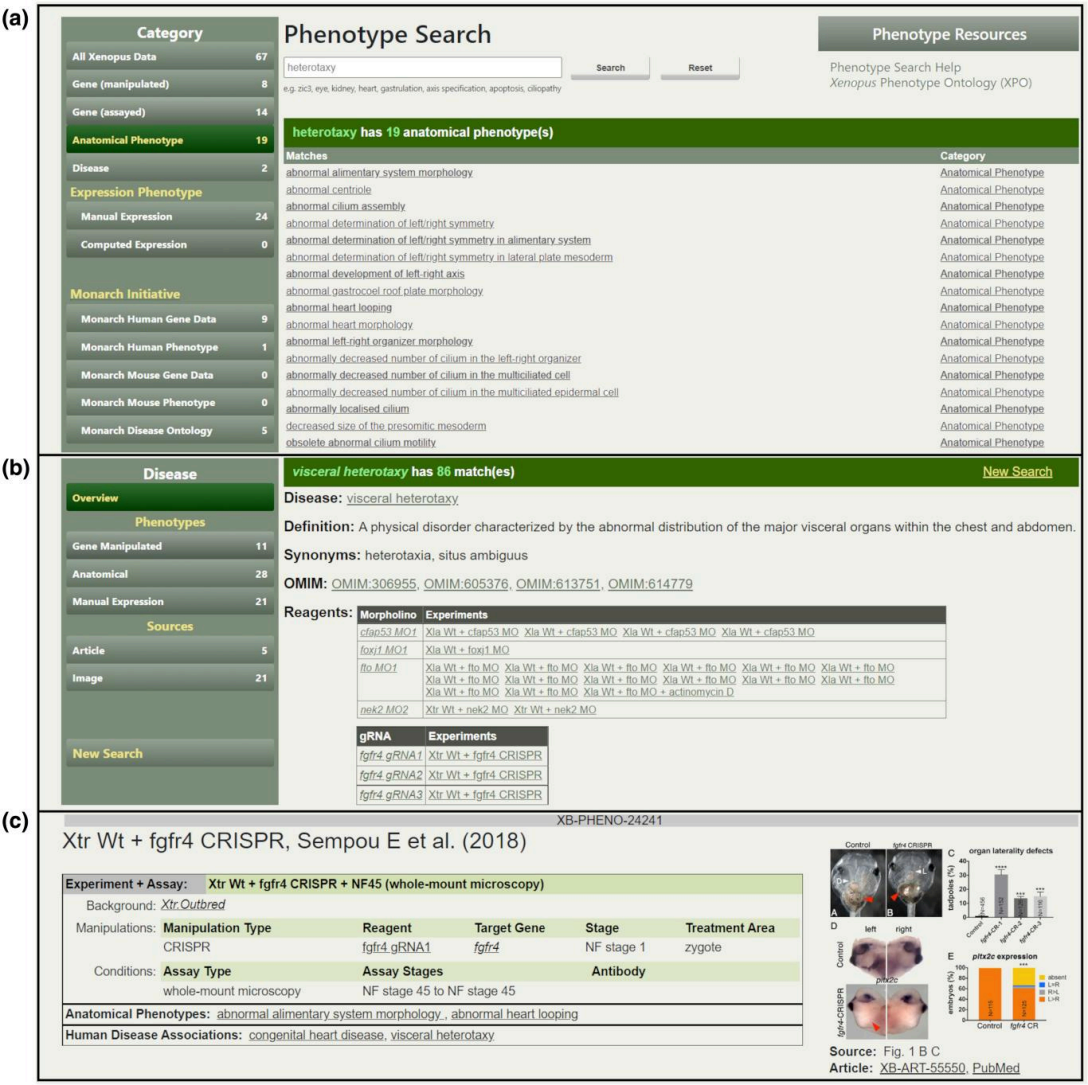
Phenotype search and XB-PHENO pages. a) The Xenbase Phenotype Search interface takes terms that are genes, anatomical entities, GO terms, and diseases and allows for partial matches. Searches can be filtered either before or after the search is run by selecting one of the categories in the left-hand sidebar. Results are also shown from the Monarch Initiative for humans and mice. In this example, results are shown for the partial DO term “heterotaxy”. b) An example result page for a Disease Ontology term search. This page shows counts for different categories of phenotype data associated with models of “visceral heterotaxy.” The default display shows descriptive information about the disease and a summary of experimental reagents used in models of the disease. c) XB-PHENO page showing anatomical phenotype and disease annotations. The layout has a heading summary with a brief experiment description and the source reference, a description of the experiment and assay details in a table, the phenotype terms and statements, and a thumbnail of the source image to the right-hand side. This example shows phenotype curation for a CRISPR experiment modeling visceral heterotaxy and congenital heart disease (Sempou *et al*. 2018). The phenotype data shown here are available at https://www.xenbase.org/entry/XB-PHENO-24241.

Phenotype data can be reached via several paths, including the dedicated Phenotype search module, research article pages, curated images, and the Phenotype tabs on Gene Pages. The Phenotype Search module provides a filterable query (Fig. 6a) for Anatomical and Expression phenotype data, genes that are experimentally manipulated or have their expression impacted, manual or computational expression phenotypes, and manual disease curation from Xenbase. The search also provides links to data for human and mouse genes and phenotypes on the Monarch Initiative website and for disease pages for the Monarch Disease Ontology (MONDO) (Shefchek *et al.* 2020).

Phenotype search result pages will aggregate the various XB-PHENOs that fit the XPO term, expression statement, or disease of interest. These pages provide different elements depending on the type of result but may include definitions, synonyms, link-outs to related resources, and a listing of experimental reagents that cause or contribute to the phenotype. Disease-related pages, for example, have links to Xenbase disease ontology pages and OMIM disease pages (Fig. 6b). Tutorial videos on the use of various elements of the phenotype search can be found at https://www.xenbase.org/entry/static-xenbase/HowTo.jsp.

Phenotype data are displayed on Phenotype pages, each of which has a unique XB-PHENO ID (Figs. 5c and 6c). Each Phenotype has a description of the experiment giving rise to the result presented as a combination of the background organism, the experimental manipulations involved, and assay conditions. A shorter summary version of this experimental and assay information is also given at the top of the table. Each phenotype has a specific source, either a figure or text reference from an article or a GEO dataset. Figure panel references are given in a specific “Source” field, while GEO-derived expression phenotypes have an associated GSE reference (Fig. 5c).

While described as “Anatomical Phenotypes,” the phenotypes in this category cover everything encompassed in the XPO, from behavioral phenotypes to ion-channel function. During image curation, the most accurate description of the phenotype described in the figure is attempted and may require several discrete XPO phenotype terms to encompass the whole phenotype.

Expression phenotypes are captured as expression statements composed of an expressed gene, an XAO anatomy term, and a PATO term describing the abnormal expression. Xenbase currently only utilizes 4 PATO terms for manual expression curation: “increased amount,” “decreased amount,” “absent,” and “mislocalized.” These statements have a form such as “increased amount *mmp8* expression in the brain” (Fig. 5c). Computational phenotypes are derived from the differential gene expression data from the Xenbase GEO pipeline. Only cases with a clear experimental intervention and control sample are used for generating these phenotype statements. We use several thresholds for identifying the differentially expressed genes displayed as Phenotype Statements, these are a 2-fold log_2_ change in expression between samples, at least one of the contributing samples needs a TPM count of greater than 1, and a false discovery rate (FDR) of less than 0.05. For computationally derived expression phenotypes, only “increased amount” and “decreased amount” are used.

Phenotype disease associations are made manually by the Xenbase curation team based on author assertions in curated articles, The author assertions need to be associated with specific phenotypes related to the disease rather than just a general gene–disease association statement.

## GO annotations

Recently Xenbase has begun to manually assign GO annotations from the primary literature using the Noctua curation platform (http://noctua.geneontology.org/). GO annotations are made available in all standard formats. GO annotations from all available sources are included in the download files, including manual annotations by the Xenbase curation team, GO Annotations from non-Xenbase sources such as UniProt and InterPro electronic annotation pipelines (Camon *et al.* 2003; Huntley *et al.* 2015; Blum *et al.* 2021), phylogeny-based annotations from the Phylogenetic Annotation and INference Tool (PAINT) project (Mi *et al.* 2019), and manual annotations from other curation projects such as AgBase (Pillai *et al.* 2012) and the University College London Cardiovascular Gene Annotation project (BHF-UCL) (Lovering *et al.* 2008). The Xenbase GO annotation files are available on the Xenbase data download site (https://download.xenbase.org/xenbase/GenePageReports/) and from the GOC (https://current.geneontology.org/annotations/). While this is intended to help provide a *Xenopus*-specific set of GO annotations, it is still heavily based on automated electronically inferred annotations so its useability for approaches such as GO term enrichment analysis has yet to be effectively tested. GO term enrichment analyses can be very sensitive to the exact composition of the reference set used (Rhee *et al.* 2008; Tomczak *et al.* 2018) making meaningful comparisons with other organisms challenging.

## Anatomy resources and the Zahn drawings

Xenbase has always provided resources related to anatomy and development for educational and research purposes. These include early embryonic fate maps, images of embryos, staging data, tables of developmental rates at different temperatures, and high-resolution images from several key reference works on *Xenopus* development, histology, and anatomy.

One of Xenbase's most heavily used resources is a collection of drawings depicting the normal stages of *Xenopus* development initially published by Nieuwkoop and Faber (1994). These are used in the laboratory to associate experiments with specific and reproducible points of embryonic development, in publications illustrating the design of embryological experiments, and in papers to illustrate the age and normal appearance of unperturbed development. Unfortunately, the link to the copyright to these images has been broken over time, and journals will not allow their inclusion in published figures. To resolve this situation, Xenbase commissioned a new set of open-access drawings of *Xenopus* embryos to generate a new comprehensive suite of reference images not only covering those in the Nieuwkoop and Faber images but also expanding the representation to include additional views and stages missed in the original resource (Zahn *et al.* 2022). These new images complement those generated by Zahn *et al.* (2017) that initiated this project with a set of drawings focused on stages and orientations relevant to craniofacial development. The new set comprises 133 high-quality illustrations spanning from fertilization to post-metamorphosis, all available under a noncommercial creative commons license and freely usable by the research community. To complement the anatomical landmarks depicted in the set of images, Xenbase also produced a comprehensive set of molecular landmarks to enable the identification of markers to verify and analyze stage-associated results with extensive links to the relevant genes and XAO anatomy and stage terms (https://www.xenbase.org/entry/landmarks-table.do). These various data are available on Xenbase (https://www.xenbase.org/entry/zahn.do) and in the original publication and accompanying poster (Zahn *et al.* 2022).

## Mutants and transgenic lines

Xenbase maintains a catalog of mutants and transgenic lines used in research and tracks their availability (where possible) from the NXR, NBRP, and EXRC (James-Zorn *et al.* 2018; Horb *et al.* 2019); we also have information for a small number of lines available from the *Xenopus laevis* Research Resource for Immunobiology (XLRRI). This catalog includes many CRISPR knockout mutants produced by the NXR (Tandon *et al.* 2017). Each line, mutant or transgenic, will have some basic information including a name based on Xenbase's established nomenclature guidelines, the species of the line, and whether the line is mutant, transgenic, or a wild-type strain. Other information is frequently provided such as Synonyms, the background strain in which the line was created, Maternal and Paternal Lines in the event of a crossed line, a description of the line and a separate description of any characteristic phenotype of the line, what gene was mutated during line generation, whether the line is isogenic; whether the line requires a Materials Transfer Agreement (MTA), whether the line was inbred or outbred, and any associated Research Resource Identifier (RRID) (Bandrowski and Martone 2016) numbers. We also provide information for ordering the lines from specific stock centers when they are available.

## *Xenopus* integration into the alliance of genome resources

Towards the end of 2022, *X. laevis* and *X. tropicalis* were added to the model organisms covered by the Alliance of Genome Resources Consortium (2020). Xenbase provides the Alliance with data for both species covering gene expression, phenotype and disease associations, *Xenopus* literature, and GO term associations. The Alliance site (http://www.alliancegenome.org/) adds a very useful layer of context by allowing *Xenopus* data to be viewed simultaneously with data for orthologs from other model organisms and humans. As part of the *Xenopus* integration, the Alliance produced a set of automatically generated gene descriptions for *X. laevis and X. tropicalis* utilizing expression, functional, and orthology data. These were produced using their established system (Alliance of Genome Resources Consortium 2022) and Xenbase hopes to have these integrated into Gene Page summary tabs in 2023. We are currently working to increase the representation of *Xenopus* data on the Alliance's site and to improve orthology calls to other species in the Alliance.

## Future directions

We are currently reprocessing all the GEO data to the new v10 genomes for both *X. tropicalis* and *X. laevis*, and these data are due to be completed in early 2023. Xenbase is currently looking into ways to incorporate single-cell RNA-Seq data into the knowledgebase to build on previous Xenbase work with high-throughput sequencing data. One requirement for this is to improve support for specific cell types in the XAO which has traditionally focused on tissue and organ-level terms. Many bioinformatics-focused web resources are developing common visualization data for single-cell data and Xenbase is investigating collaborations with other resources or the creation of a Xenbase-specific viewer.

Xenbase is working to maintain and improve our communications with other online resources, such as CRISPRScan (Moreno-Mateos *et al.* 2015), whose genome-wide CRISPR target site prediction data we currently provide in JBrowse, the Monarch Initiative, and the Alliance of Genome Resources. This all contributes to making *Xenopus* data deeply integrated into the corpus of biomedical knowledge and, therefore, accessible to the broader scientific community.

## Data Availability

All data in this article are available at https://www.xenbase.org or at the given URLs.
